# Evaluation of Skin Irritation and Acute and Subacute Oral Toxicity of* Lavandula angustifolia* Essential Oils in Rabbit and Mice

**DOI:** 10.1155/2019/5979546

**Published:** 2019-01-27

**Authors:** Awol Mekonnen, Solomon Tesfaye, Selam G. Christos, Kassahun Dires, Tizazu Zenebe, Nigus Zegeye, Yoseph Shiferaw, Ermias Lulekal

**Affiliations:** ^1^College of Medicine, Institute of Health Science and Medicine, Debre-Berhan Universty, P.O. Box 445, Debre-Berhan, Ethiopia; ^2^School of Medicine, Institute of Health Science and Medicine, Jijiga University, P.O. Box, 1020, Jijiga, Ethiopia; ^3^Chemistry Department, College Computational Sciences and of Natural, Debre-Berhan University, P.O. Box 445, Debre-Berhan, Ethiopia; ^4^Plant Biology and Biodiversity Management Department, College of Computational Sciences and Natural, Addis Ababa University, Private P.O. Box 34731, Addis Ababa, Ethiopia

## Abstract

*Lavandula angustifolia* is used in traditional and folk medicines of Ankober District, North Central Ethiopia, for the treatment of several livestock and human disorders. This toxicity study aimed to investigate* L. angustifolia* essential oil oral toxicity in mice and skin irritation in rabbit.* L. angustifolia* essential oil was analyzed using gas chromatography-mass spectrometry methods and showed predominance of Eucalyptol (52.36%), Camphor (11.91%), gamma-terpinene (8.775%) and endoborneol (7.585%). Limit test at 2000 mg/kg dose was used for* L. angustifolia* essential oil acute toxicity test and revealed LD_50_ value was higher than 2000 mg/kg. For subacute toxicity study 2000mg/kg was given orally to each mouse for 21 days. The result demonstrated no significant changes (p > 0.05) in the body weights, and biochemical parameters, gross abnormalities, water, and food intake were observed. No macroscopic changes were seen in the histopathology analysis of kidneys and livers. For skin irritation test shaved rabbit skin was treated with 10% ointment formulation. Ointment of* L. angustifolia* oil did not affect mice skin. Generally, this toxicity study demonstrated that* L. angustifolia* essential oil is nontoxic.

## 1. Introduction

Of all the essential oils (EOs) used commercially, lavender (*Lavandula angustifolia*) oil appears to be one of the most popular.* L. angustifolia*, is a frost hardy aromatic plant of the Lamiaceae family that has many pretty cultivars, habit, and blossom color. It widely distributed in the Mediterranean area. Though different factors affects the* L. angustifolia *EO composition, several phytochemical studies revealed that linalool, linalyl acetate, camphor, endoborneol, and 1,8 cineole are the major components [1, 2]. Lavender EO have numerous medicinal value [3]. The earliest therapeutic use of* L. angustifolia* can be traced back to Roman and Greek times [4]. Several experimental studies on* L. angustifolia *testified its Soporific [5], antispasmodic [6], anticonvulsive (Yamada* et al*., 1994), anesthetic (Ghelardini et al., 1999), diuretic [7], antidepressant and anxiolytic [8], dyslipidemia [9], cardioprotective [10], analgesic and anti-inflammatory [11], wound healing [12], insecticidal [13], antiproliferative, antimicrobial, and antioxidant [14] activities.

The* L. angustifolia* is used in traditional and folk medicines of Ankober District, North Central Ethiopia for the treatment of several livestock and human disorders. In several countries including Ethiopia, many a times the beneficial claims made for a herbal based drug are not backed by sufficient preclinical and animal toxicity data. Toxicological studies help to make decision whether a new substance should be adopted for clinical use or not [15]. In cosmetic industry and drugs for topical application, evaluation of irritancy potential of any formulations/chemicals to human skin is a necessity [16].

Evaluation of toxic properties of a substance is crucial when considering for public health protection because exposure to chemicals can be hazardous and results to adverse effects on human being. In practice, the evaluation typically includes acute, subacute, subchronic, chronic, carcinogenic and reproductive effects [17]. Though* L. angustifolia* is widely used by the Ethiopian population for treatment of several diseases, no systematic evaluation of its toxic effects has been carried out. This toxicity study is primarily aimed to assess the safety of* L. angustifolia* upon 21-day repeated oral dosing of extract in mice.

## 2. Methods

### 2.1. Plant Collection and Authentication

Fresh leaves and flowers of* L. angustifolia* were collected from Ankober Project Nursery Site in North Shoa Zone, Amhara Regional State, Ethiopia. The plant materials were authenticated by the National Herbarium in Addis Ababa University, Ethiopia.

### 2.2. Extraction of* L. angustifolia* Essential Oil

Samples were air dried under shade at room temperature for ten days. Dried* L. angustifolia* leaves and flowers were extracted by a steam distillation for 3hrs. The percentage yield found was 0.45% and 0.24% volume by mass for dried leaves and flower heads respectively. In this toxicity study, EO extracted from the leaf part was used.

### 2.3. Analysis and Compound Identification of* L. angustifolia* Essential Oil

*L. angustifolia* EO analyses were carried out using a gas chromatography/mass spectrometry system (GC-MS). GC-MS analysis was performed by using Agilent 5977A (Agilent Technologies, Germany). GC was equipped with a 30 m HP-5 capillary column with 0.25 mm internal diameter and 0.25 *μ*m film thickness. The helium carrier gas had a delivery rate of 1 ml/min. The injector temperature was held at 250°C and the detector temperature was held at 250°C. The injection volume was 1 *μ*l. The split ratio was 40:1. The temperature program was as follows: the initial temperature was held at 50°C for 6 min and then increased to 76°C at a rate of 4°C/min for 5 min, then increased 190°C at the rate of 4°C/min, then increased to 270°C at the rate of 30°C/min and held for 7 min. The constituents were identified by comparison of their mass spectra with those of NIST 14 & Wily library data for the GC-MS system. Percent composition of each compound was calculated from peak area using normalization method.

### 2.4. Ointment Formulation

10% (w/w) ointment of* L. angustifolia* was formulated by incorporating 10g of EO in 100g petroleum jelly for skin irritation assessment. Ointment was examined for their physical properties including viscosity, spreadability, extrudability, homogeneity, and physical appearance using methods described in Bora et al. [18] and Nair et al. [19].

### 2.5. Experimental Animals Husbandry

Male and female mice (Swiss albino) of 10-12 weeks old from School of Pharmacy, Addis Ababa University and female rabbits (New Zealand) weighing 1.4-2.3 kg from the Ethiopian Public Health Institute were used. Animals were housed in standard cage in a ventilated room under room temperature of 25 ± 2°C with 12hr light/dark cycle. Animals were acclimatized for minimum of 5 days to the laboratory conditions prior to experimentation and were fed with standard food pellets and water* ad libtum*. Debre-Berhan University ethical committee approved this study and the experimental animals were cared following the ILAR [20] guideline.

### 2.6. Skin Irritation Test

Skin tolerance tests were done using the Organization for Economic Cooperation and Development guidelines [21] with slit modification. Twenty four hours before the experiment, fur from the backs of all rabbits was clipped on different site (Figure 2). Half a gram of 10%* L. angustifolia* EO ointment formulation was evenly and gently applied in a test site while untreated skin areas serve as control. The test sites were then examined critically at 1hr after removing the test material and at 24hrs, 48 hrs, 72 hrs, 7th and 15th day for dermal reaction using Draize scoring criteria [21, 22].

### 2.7. Acute Toxicity Study

All animals were deprived of food for 8 hours prior to commencing the experiment. Dose of 2000 mg/kg body weight* L. angustifolia* EO was administered orally at to one female mouse [23]. After two days, the same dose was administered orally to four female mice increasing the number of treatment animals to five. The second group of 5 female mice, negative control group, was administered with equal volume of saline. Each mouse was observed critically during the first four hrs, periodically for the first 24 hrs and once a day for 14 days. During this period the activities related to motor-muscle coordination and central and autonomic nervous system were analyzed.

### 2.8. 21-Day Repeated Dose Toxicity Test

Repeated dose toxicity test of the* L. angustifolia *EO was conducted according to OECD 407 guideline [24]. Twenty (10 male and 10 female) mice were randomly divided into two groups each having five male and five female mice. Dose of 2000 mg/kg* L. angustifolia *EO and saline daily once for 21 days were given to treatment and control groups respectively. The body weight of all mice and feed consumption of each cage were recorded weekly and also subjected to daily observations for mortality, behavioural changes, and possible symptoms of humane end point during the 21-day experimental period.

#### 2.8.1. Histopathological Examination and Biochemical Parameters

Blood samples for biochemical analyses were taken from carotid artery at the 22th day from both treated and untreated groups. The serum was separated and the amount of creatinine, alkaline phosphatase (ALP), serum glutamic-oxaloacetic transaminase (SGOT-A), and serum glutamic-pyruvic transaminase (SGPT-L) were measured. Each mouse was euthanized for gross pathological examinations of major internal organs. Kidney and liver of all animals were conserved in 10% buffered formalin after measuring the weight for histopathological examination. Tissues were trimmed in 5*μ*m thickness and stained by haematoxylin and eosin. Tissue section was examined under light microscope, photomicrographs were taken at ×4 and ×40 [25].

### 2.9. Statistical Analysis

Results are reported as mean ± standard errors of the mean (SEM). Independent samples t test was used for comparison between two groups. Differences were considered significant at P < 0.05.

## 3. Results

### 3.1. Chemical Composition of* L. angustifolia* Essential Oil

**Figure 1:**
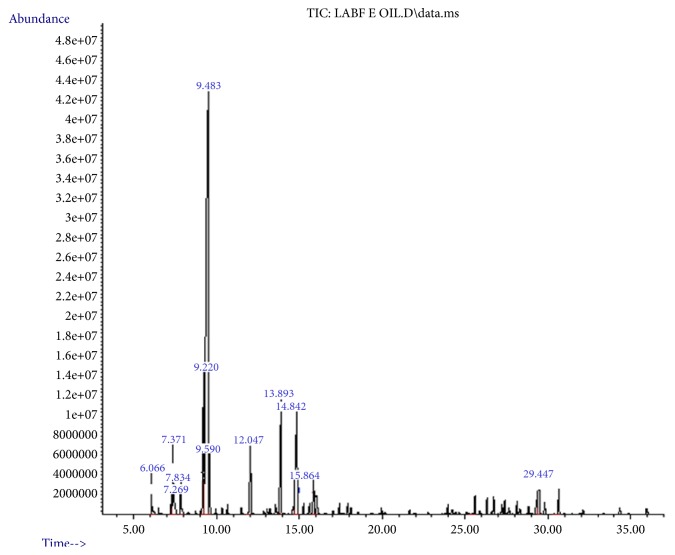
Chromatogram of* L. angustifolia *essential oil.

The yield of* L. angustifolia* leaves EO was 0.45%v/w. The four major components of the* L. angustifolia *EO were eucalyptol (52.362%), camphor (11.915%), gamma-terpinene (8.775%) and endoborneol (7.585%) (Figure 1). The result from GC-MS analysis revealed that 63 components in the oil and 12 of them were identified representing 98.401% of the total composition (Table 1).

### 3.2. *L. angustifolia* Essential Oil Ointment Characteristics

The physicochemical characteristics of* L. angustifolia* EO ointment formulation were depicted in Table 2.* L. angustifolia* EO ointment was homogenous, smooth, nongritty, and white in colour with characteristic odour of the EO.* L. angustifolia* EO ointment showed several favorable properties such as easily spreadable, easily removable, easily extrudable, and acceptable viscosity.

### 3.3. Skin Irritation Assessment

**Figure 2:**
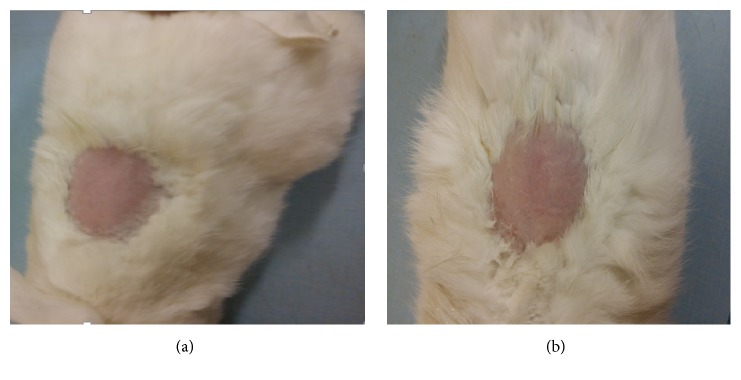
Photo of rabbit skin before (a) and one hr after removing the test ointment (b).

Table 3 showed skin tolerance test findings. Rabbits showed no irritation signs or skin edema after treatment with 10% ointment of* L. angustifolia* EO. The treated skin was intact; no inflammation and erythema compared to untreated site (Figure 2). Edema and erythema score was “0” in each rabbit at any time of the observation after removing the test material. This demonstrated that the skin Primary Irritation Index score was 0.

### 3.4. Acute Toxicity Study

The results revealed that the single dose treatment with* L. angustifolia* EO by oral route at 2000 mg/kg dose did not cause death in female mice throughout the study period. No treatment related toxicity signs were noted. Moreover, EO produced no change in the skin, eyes, diarrhea, salivation, and behaviour patterns of mice. Hence, lethal dose (LD_50_) for oral route of* L. angustifolia* EO was estimated to be higher than 2000 mg/kg.

### 3.5. Repeated Dose Oral Toxicity Assessment

#### 3.5.1. General Observation, Body Weight, and Food Consumption

Dose of 2000 mg/kg daily oral administration of* L. angustifolia* EO for 21 days did not cause any treatment-related toxicity signs, behavioural changes, and death in both sexes of mice. The body weight measurements showed suppressed weight gain in female treated mice (22.5±10.6%) compared to treated male mice (87±14.4%) and control (Table 4). Nevertheless, the difference was not significant statistically. Similarly, the food consumption of female treated mice was lower compared to treated male mice and untreated group. Yet, the difference was not significant statistically.

#### 3.5.2. Organ-Body Weight Ratio

The necropsy showed no abnormal findings in the male and female EO treated and control groups. The differences in relative organ weight of liver and kidneys recorded between the treatment and the control groups were not significant statistically (Table 5). However, the relative organ weight of female treated mice was slightly higher than untreated group. On contrary, the relative organ weight of male treated mice was slightly lower than control. But, no difference in the parameters related to liver and kidney toxicity was observed in serum biochemical and histopathological examinations. Hence, the aforesaid changes were not considered toxicologically significant

#### 3.5.3. Liver and Kidney Function Indices

Biochemical analysis (Table 6) showed no significant difference (*P>0.05*) in any of the parameters analyzed in either control or treatment group of both female and male mice after 21 days oral administration of the EO. Nonetheless, the amounts of ALPL, SGPT-L, and SGOT-A were a bit higher in female treated mice except CREAT-A as compared with untreated group. The amount of all measured biochemical parameters was lower in treated male mice except ALPL as compared with control mice. Yet, this difference is not significant statistically.

### 3.6. Histopathology Examination

**Figure 3:**
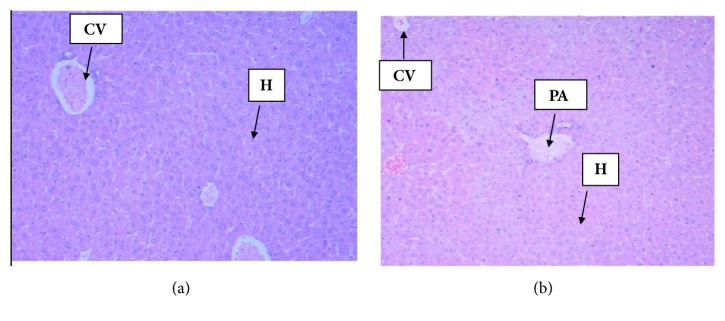
Photomicrographs (H&E, magnification x 4) of liver histology. (a) Control group liver section showed normal hepatocytes; (b) Section of liver from* L. angustifolia* EO (2000 mg/kg) treated group depicted normal hepatocytes architecture after 21 days of administration. CV=Central Vein, H= Hepatocytes, PA=Portal Area.

**Figure 4:**
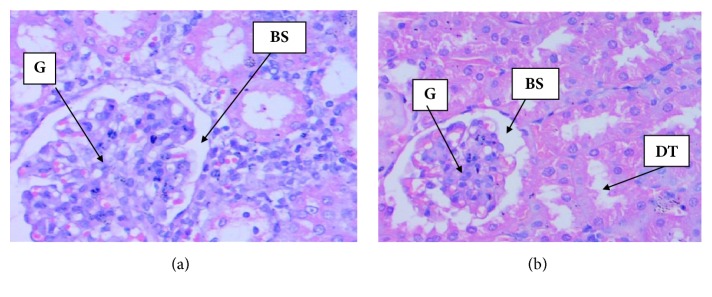
Photomicrographs (H&E, magnification x 40) of kidney histology. (a) Control group kidney section revealed normal glomeruli and tubules; (b) Section of kidney from* L. angustifolia* EO (2000 mg/kg) treated group exhibited normal glomeruli and tubules after 21 days of administration. G=Glomerulus, BS=Bowman's Space, DT= Distal Convoluted Tube.

Figures 3 and 4 displayed histopathological features of kidney and liver of treated and untreated group. No histopathological changes were noted both in the treated and in the untreated group. The result revealed normal periportal, normal hepatocyte morphology, with no evidence of inflammation and necrosis. Correspondingly, all sections from the kidney exhibited normal glomerular and unremarkable typical tubule interstitial parenchyma, with no hyaline changes or vascular necrosis.

## 4. Discussion

*L. angustifolia *is characteristic of strong fragrance, indicating that there are rich aromatic compounds. The oil is generally composed of complex mixture of monoterpene, biogenetically related phenols, and sesquiterpenes (Yazdani et al., 2013). The EO obtained in this study was rich in monoterpene and phenols. GC-MS analysis showed that eucalyptol, camphor, gamma-terpinene, endoborneol, and beta- pinene are the major components, which suggests that these compounds may play major roles in the biological activities. In a previous phytochemical study with this plant showed the presence of the same major components of* L. angustifolia *EO, however, in different concentrations [1, 26]. The chemical composition of EOs may show variations due to geographic origin, geographic conditions, climate, seasonality, plant part used, stage of the plant, extraction, and detection methods [1, 27].

Several scientific papers reported plentiful therapeutic uses of* L. angustifolia* as an alternative medicine effective for a wide range of diseases [28, 29]. Hence, investigation of safety profile of this plant is used to guide the management of its applications and usage in herbal preparations. Toxicity studies in appropriate animal models are commonly used method to assess potential health risks in humans [30]. In this toxicity study, acute oral treatment of female mice with* L. angustifolia* EO at 2000 mg/kg had no effects on mortality, examined clinical signs, or overall observation. Therefore, the approximate median lethal oral dose was determined to be higher than 2000 mg/kg. According to the Globally Harmonised Classification System for Chemical Substances and Mixtures of acute systemic toxicity, the* L. angustifolia* EO was assigned class 5 status (2000 mg/kg<LD50<5000 mg/kg) which was the lowest toxicity class [21]. The finding of this toxicity assessment was in agreement with study conducted by Silva et al., [11] who reported that LD_50_ value was 3.55g/kg.

Knowledge about skin sensitization potential is an explicit need for both hazard and risk assessment. Proper skin sensitization data of the individual chemicals is essential, especially when dermal contact is intended [31]. Dermal irritation test of* L. angustifolia* EO formulation is essential toxicity screening which provides a fundamental characterization of the potential hazards to skin. The prepared ointment was evaluated for its skin irritant effect, where no erythema or edema was observed with PII equal to 0 indicating that the prepared herbal formulation is safe for topical use. This finding was in line with study done by Opdyke, [32] who reported that undiluted lavender oil was not irritant when applied to the backs of hairless mice or pigs but was slightly irritant on intact or abraded rabbit skin under occlusion for 24 hours.

Although acute toxicity helps to determine the toxicity nature of substances, it is important to assess subacute oral toxicity profile because it helps to evaluate the morphological and physiological changes in organs after repeated administration [33, 34]. In oral toxicity assessment the body weight change is sensitive and indicative marker for toxicity sign [35]. No significant changes in the body weight were observed in treated mice compared to untreated group after 21 days administration of* L. angustifolia* EO. However, a slight retardation of the body weight gain and food intake was noticed in the female mice, but no effects were evident from histopathology and clinical chemistry. As optimal supplements intake is important to the physiological wellbeing of the animal, it is also important to determine the water and food intake during toxicological study [34, 36]. In this toxicity study, water and food intake were not affected by the administration of* L. angustifolia* EO indicating that no interruption in the metabolism of carbohydrate, protein, and fat [34]. Changes in organ weights are a clear indicative of damage caused by the substance under test [25]. No gross abnormality was found in the organs at necropsy and did not produce organ swelling, atrophy, or hypertrophy.

Results of biochemical analysis demonstrated that* L. angustifolia* EO did not produce toxic effect. Serum biochemicals like ALP, SGPT, and SGOT still keep the gold standards for the assessment of liver injury and have been served as biomarkers of choice for decades [37, 38]. Examination of the biochemical parameters of the blood serum showed no significant changes in EO treatment group compared to control (Table 6), which indicates absence of any harmful effects on liver. Measurements of urea and creatinine levels in the blood are usually performed to evaluate kidney function [39]. In renal toxicity, these two parameters are usually markedly increased higher than the normal values. In this toxicity study creatinine levels were not significantly different between treated and untreated group (p>0.05), which indicated* L. angustifolia *EO had no toxic effects on the kidneys. The findings were further established by the histological study of the organs shown in Figures 3 and 4. The histological examination is the golden standard for evaluating treatment related pathological changes in organs and tissues. Histopathological examination of kidneys showed normal features of renal tubules and glomeruli suggesting that normal renal function of the treated and untreated group. Liver histopathology showed normal morphology of hepatic cells, central vein, and portal triads indicating normal liver function in both treated and untreated group. The assessment of pathological alteration in the organs of treated animals, both macro and microscopically, is the basis of a safety assessment [40]. Absence of any histopathological change in this toxicity study which was proved to be consistent with biochemical analyses confirms the safety of using* L. angustifolia* EO for 21 day repeated administration.

## 5. Conclusion

The present result demonstrates the absence of acute and subacute toxicity of* L. angustifolia* EO at 2000 mg/kg. Skin tolerance assessment on rabbit depicted* L. angustifolia* EO ointment was not irritant. In the subacute toxicity study, there were no toxicologically significant changes in all measured parameters. In conclusion, to the best of our knowledge, this toxicity study may represent the first study that demonstrates absence of 21-day repeated dose toxicity of* L. angustifolia* EO in mice at 2000 mg/kg.

## Figures and Tables

**Table 1:** * L. angustifolia* leaves oil chemical composition.

**RT**	**Constituent **	%** Composition **	**MF **	**Mass Fragments **
6.070	Alpha-pinene	3.811	C_10_H_16_	41,43,53,67,74,77,89,90,105,121,136

7.273	Beta-phellandrene	0.381	C_10_H_16_	41,43,53,65,69,77,79,91,93,,105,115,121,136

7.376	Beta- pinene	4.967	C_10_H_16_	41,43,53,67,69,77,86,91,93,107,121,132,136

7.837	Beta-myrcene	1.949	C_10_H_16_	41,43,51,53,55,59,67,69,79,86,91,93,107,115,121,136

9.221	Gamma-terpinene	8.775	C_10_H_16_	43,51,55,65,59,69,77,89,91,93,105,121,136

9.485	Eucalyptol	52.362	C_10_H_18_O	41,43,53,55,58,65,67,69,71,79,81,84,93,96,108,111,125,136,139,154

9.583	Trance-beta-ocimene	1.040	C_10_H_16_	41,43,51,53,67,74,79,81,91,93,96,105,107,121,136

12.040	Linalyl acetate	1.344	C_12_H_20_O_2_	41,43,45,51,53,60,65,55,69,71,80,83,87,90,99,107,121,136,151,167

13.919	Camphor	11.915	C_10_H_16_O	41,43,45,51,53,56,66,67,69,77,81,83,93,95,108,119,124,137,152

14.816	Endo-borneol	7.585	C_10_H_18_O	41,43,55,57,61,65,67,71,79,83,93,95,110,121,126,139,152

15.861	Alpha-terpineol	1.874	C_10_H_18_O	41,43,45,55,59,65,67,71,79,81,88,93,95,107,115,121,136,139

29.483	Gamma-muurolene	2.398	C_15_H_24_	41,55,69,79,93,105,119,133,147,161,175,189,204

Total		98.401		

RT= Retention time and MF= molecular formula.

**Table 2:** Physicochemical evaluation of* L. angustifolia* ointment formulation.

**Formulation**	**Color**	**Viscosity (cP) at 100 rpm**	**Spreadability (g.Cm/min)**	**Extrudability (g)**
		**(Mean±SD)**	**(Mean±SD)**	** (Mean±SD)**
*L. angustifolia* oil ointment	White	8164.3±11.9	2233.5±206.2	0.136±0.015

**Table 3:** Score of edema and erythema after removing the test formulation.

Skin reaction score of 10%* L. angustifolia* essential oil ointment at various time interval (3 treatment site)												

Reaction	1hr		24hrs		48hrs		72hrs		7th day		15th day	
	**Con**	**Trt**	**Con**	**Trt**	**Con**	**Trt**	**Con**	**Trt**	**Con**	**Trt**	**Con**	**Trt**

Erythema	0	0	0	0	0	0	0	0	0	0	0	0
Edema	0	0	0	0	0	0	0	0	0	0	0	0

**Primary Irritation Index (PII) = 0/3, PII=0.**

**Based on PII irritation category for 10**%** L. angustifolia EO ointment is Negligible.**

**Table 4:** Body weight and food consumption of mice orally treated with *L. angustifolia *EO for 21 days.

		**Control**	**LA 2000 mg/kg**
**Female**			
	Initial Body Weight (g)	20	20
	Final Body Weight (g)	33.5±2.4	24.5±2.1
	Body Weight Gain (%)	67.5±11.9	22.5±10.6
	Food Intake (g/week)	171.3±37.6	65.5±54.4
**Male**			
	Initial Body Weight (g)	20	20
	Final Body Weight (g)	28.5±0.7	37.4±2.9
	Body Weight Gain (%)	42.5±3.54	87±14.4
	Food Intake (g/week)	122±48.1	121.5±53.1

Values are expressed as mean ± SEM, n = 5 animals/group, p > 0.05 (Independent-samples T test), and LA=* L. angustifolia*.

**Table 5:** Relative organ weight (g/100 g of body weight) of mice orally treated with *L. angustifolia *EO for 21 days.

		**Control**	**LA 2000 mg/kg**
**Female**			
	Liver (g)	5.6±0.9	7.2±0.2
	Kidney (g)	1.5±0.2	1.6±0.14
**Male**			
	Liver (g)	8.06±0.6	6.2±1.4
	Kidney (g)	2.1±0.1	1.7±0.2

Values are expressed as mean ± SEM, n = 5 animals/group, p > 0.05 (Independent-samples T test), and LA=* L. angustifolia*

**Table 6:** Effect of *L. angustifolia* EO on biochemical parameters of orally treated mice.

		**Control**	**LA 2000 mg/kg**
**Female**			
	CREAT-A	0.8±0.1	0.8±0.1
	SGOT-A	194±36.8	204±14.1
	SGPT-L	39.7±12.7	45.5±9.2
	ALPL	289.3±77.2	352±4.2
**Male**			
	CREAT-A	0.8±0.1	0.76±0.1
	SGOT-A	256.5±68.6	210.6±57.4
	SGPT-L	65±24.04	58.4±13.9
	ALPL	197.5±92.6	260±212.2

Values are expressed as mean ± SEM, n = 5 animals/group, p > 0.05 (Independent-samples T test), and LA=* L. angustifolia*

## Data Availability

The datasets generated during and/or analysed during the current study are available from the corresponding author on reasonable request.
